# TACR2 is associated with the immune microenvironment and inhibits migration and proliferation via the Wnt/β-catenin signaling pathway in prostate cancer

**DOI:** 10.1186/s12935-021-02126-0

**Published:** 2021-08-07

**Authors:** Wang Jianfeng, Wang Yutao, Bi Jianbin

**Affiliations:** Department of Urology, The First Hospital of China Medical University, Shenyang, Liaoning People’s Republic of China

**Keywords:** Tachykinin receptor 2 (TACR2), Immune microenvironment, Wnt/β-catenin signaling pathway, Prostate cancer

## Abstract

**Background:**

The tachykinin receptor 2 (TACR2) is encoded by the tachykinin receptor correlation gene. Recent microarray analysis for prostate cancer suggests that TACR2 expression is associated with clinical phenotype and disease-free survival among patients with prostate cancer.

**Results:**

TACR2 protein levels were lower in prostate cancer tissues than in adjacent normal prostate tissue. TACR2 expression significantly correlated with clinical stage, Gleason scores, and survival outcomes. TACR2 expression positively correlated with mast cells and negatively correlated with M2 macrophages. Overexpression of TACR2 promoted the migration and proliferation of prostate cancer cells by regulating the Wnt signaling pathway.

**Conclusions:**

The TACR2-Wnt/β-catenin signaling pathway is critical in prostate cancer. TACR2 may affect tumor cells’ occurrence and development by changing the content of immune cells in the tumor microenvironment. These findings suggest that TACR2 may be a candidate molecular biomarker for prostate cancer therapy.

**Supplementary Information:**

The online version contains supplementary material available at 10.1186/s12935-021-02126-0.

## Background

Prostate cancer is the second most common malignant tumor in men and the fifth most common cause of cancer death worldwide. Although its mortality has decreased in recent years, more than 300,000 men still die of prostate cancer every year; especially in developed countries, prostate cancer prevalence remains very high [1–3]. Prostate-specific antigen (PSA) is a kallikrein-like serine protease secreted by prostatic epithelial cells. Its serum expression levels can be used to screen for prostate cancer. Nevertheless, the utility of PSA is limited and controversial [4–6]. Therefore, new biomarkers are urgently needed to measure the progression of prostate cancer more accurately.

In a previous study, we established and validated a prognostic risk score formula called CMU5 based on the expression of five genes: *FAM72D*, *ARHGAP33*, *TACR2*, *PLEK2*, and *FA2H*; the signature reliably predicted prostate cancer outcome [7]. Among these five genes, we focused on *TACR2*, which encodes tachykinin receptor NK2, one of the three tachykinin receptors that belong to the G-protein-coupled receptor superfamily [8]. It is not only expressed in the peripheral and central nervous system but also in some peripheral tissues [8, 9]. Tachykinins interact with tachykinin receptors and participate in critical physiological processes of the respiratory, cardiovascular, immune, endocrine, gastrointestinal, and genitourinary systems, as well as other peripheral organ systems [10, 11]. Tachykinins are involved in the development of many diseases, including cancer. Abnormal expression of tachykinin receptor coding genes was found in breast cancer, colon cancer, and other tumors. Nevertheless, the role of TACR2 in cancer progression remains unclear [11–13].

The Wnt signaling pathway includes the canonical Wnt/β-catenin signaling pathway, the non-canonical Wnt/Ca^2+^ signaling pathway, and the Wnt/planar cell polarity signaling pathway [14]. The classic Wnt/β-catenin signaling pathway is the most characterized Wnt pathway [15]; it is involved in various cellular functions, including cell proliferation, stem cell self-renewal, and organogenesis [15–17]. The Wnt/β-catenin signaling pathway is closely related to the occurrence and development of breast cancer, colon cancer, ovarian cancer, prostate cancer, and other malignant tumors. It is essential for the migration, proliferation, angiogenesis, and invasion of cancer cells [16–20]. In this study, we determined the effect of TACR2 on the immune microenvironment of prostate cancer by examining the content of immune cells. In addition, we found that TACR2 may affect the migration and proliferation of prostate cancer cells by regulating the Wnt/β-catenin signaling pathway. These findings suggest that TACR2 may be used as a marker of prostate cancer development and may become a potential therapeutic target.

## Results

### TACR2 was downregulated in tumor tissue

**Fig. 1:**
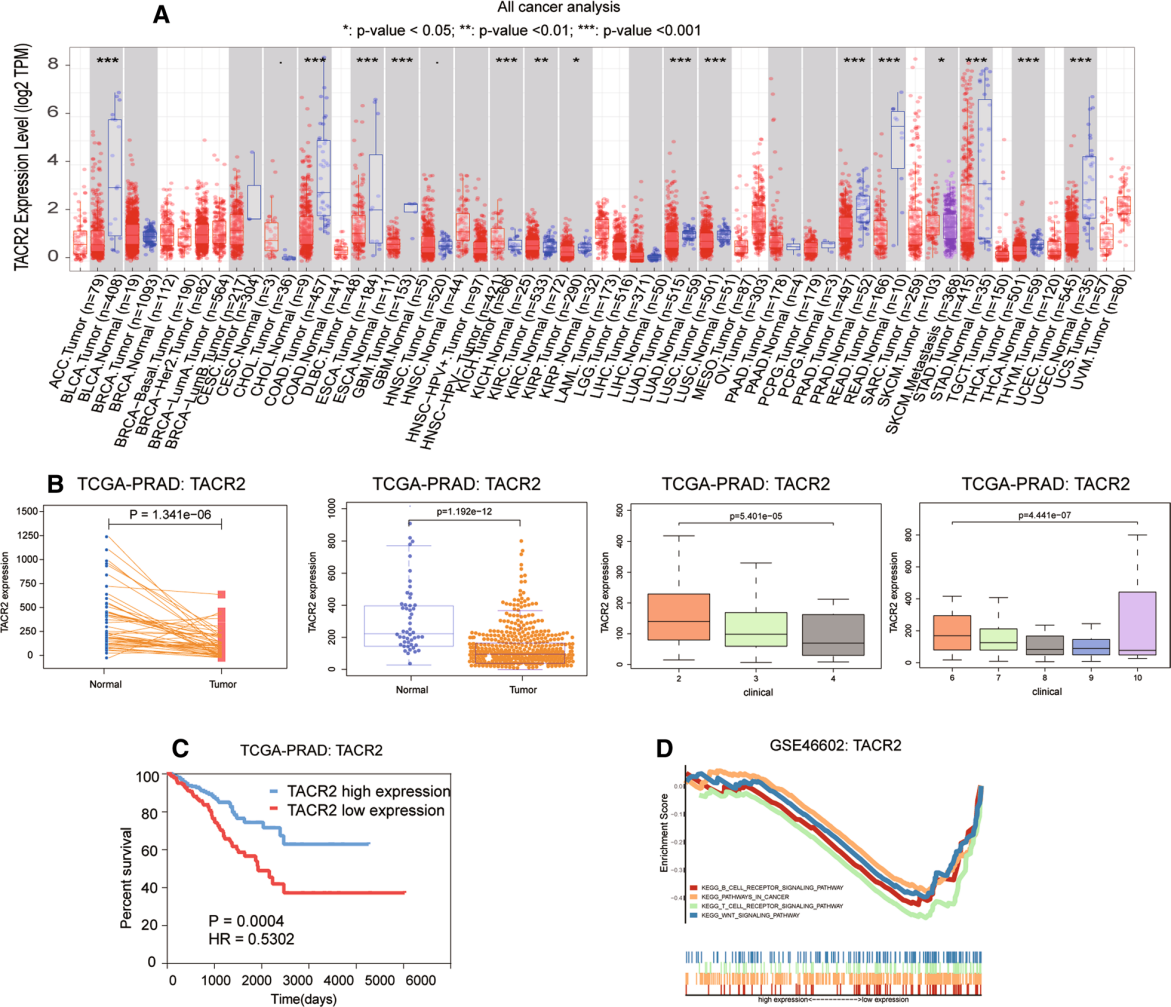
**A** The expression of TACR2 in all cancers was obtained using the TIMER database. Among them, in kidney chromophobe, kidney renal clear cell carcinoma, and kidney papillary cell carcinoma (KIRP), the expression levels of TACR2 in tumor tissues were lower than that in normal tissues P values were all lower than 0.05, suggesting statistically significant results. **B** Pair design was performed in TCGA-PRAD to calculate the expression of TACR2 in tumor tissues and normal tissues of the same patient (*P* = 1.341e−06). **C** The expression of TACR2 in tumor tissues and normal tissues of different patients was calculated by randomization in TCGA-PRAD (*P* = 1.192e−12). **D** Expression of TACR2 in the three clinical stages of PRAD (*P* = 5.401e−05). **E** The expression of TACR2 in different Gleason score groups of PRAD (*P* = 4.441e−07). **F** Survival analysis of the group with high expression of TACR2 and the group with low expression of TACR2 showed that the group's survival rate with high expression of TACR2 was higher (*P* = 0.0004, HR = 0.5302). **G** In GSE46602, TACR2 is enriched in the B cell receptor signaling pathway, cancer pathways, the T cell receptor signaling pathway, and the Wnt signaling pathway

To explore the expression pattern of TACR2, we used the TIMER database to determine that TACR2 expression was significantly downregulated in a variety of cancer tissues. Of these, TACR2 expression was significantly downregulated in prostate cancer, clear cell renal cell carcinoma, chromophobe tumor, and papillary renal cell carcinoma (Fig. 1A). We performed a paired t-test and clinical correlation analysis in TCGA-PRAD. We found that TACR2 expression levels were low in tissues adjacent to prostate cancer and were higher in patients with higher clinical stages and higher Gleason scores (Fig. 1B, *P* < 0.001). In the survival analysis with disease-free recurrence as the follow-up endpoint, TACR2 high-expressing prostate cancer patients had better survival outcomes than low-expressing patients (Fig. 1C, *P* < 0.001).

### TACR2 immune correlation

**Fig. 2:**
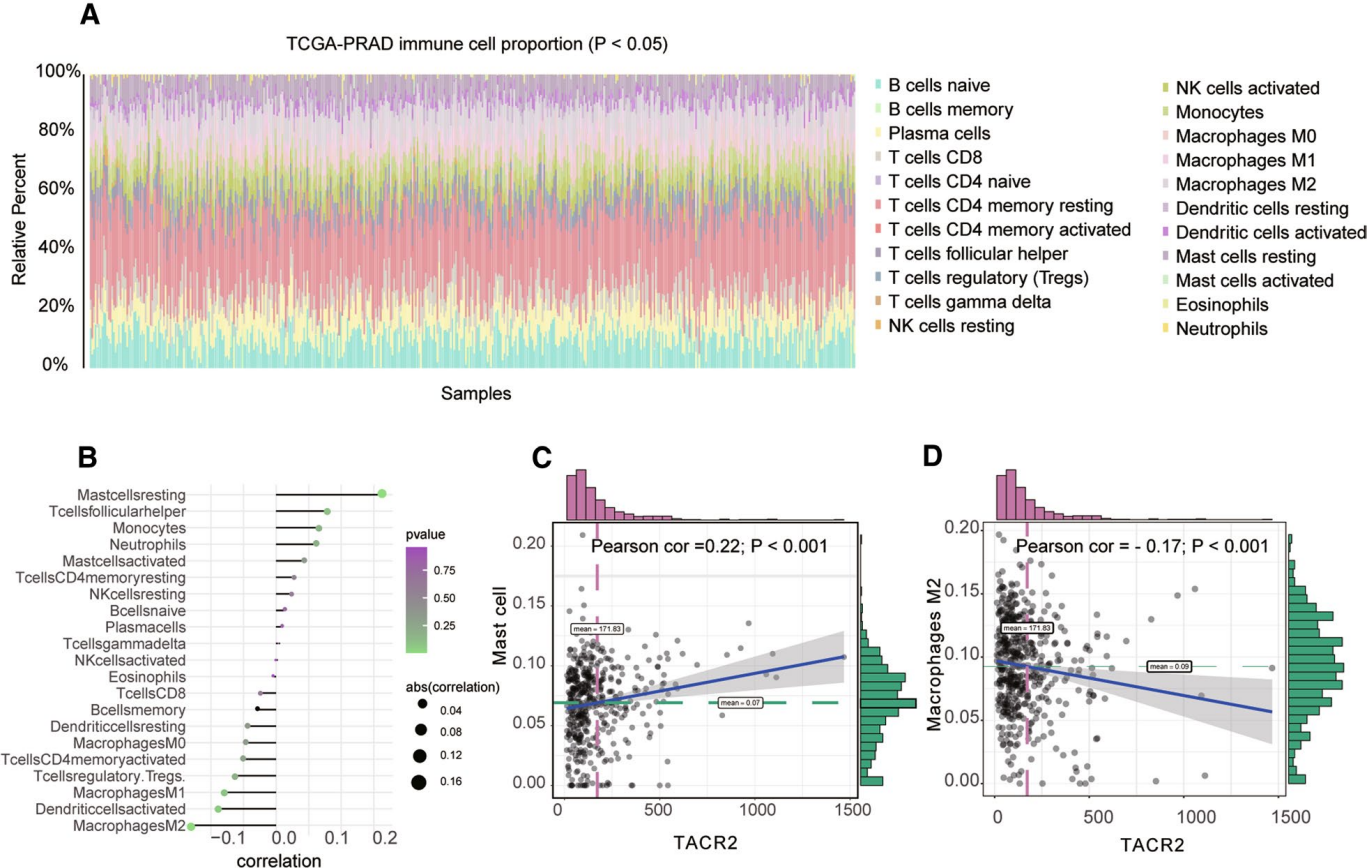
**A** Panoramic view of immune infiltration in TCGA-PRAD. **B** Correlation between TACR2 and various immune cells in the immune microenvironment of PRAD. **C** TACR2 expression was most positively related to resting mast cells (Pearson COR = 0.22; *P* < 0.001). **D** TACR2 was most negatively related to M2 macrophages (Pearson COR = − 0.17; *P* < 0.001)

In GSE46602, we divided the expression matrix into two groups according to the median expression of TACR2. We then compared the differential pathways in the two sets of samples according to the GSEA analysis strategy. The T cell receptor and Wnt pathways were significantly enriched (Fig. 1D). These findings suggest that TACR2 may be closely related to these two pathways. Therefore, to conduct a detailed analysis of these two pathways, we measured the immune cell content in TCGA-PRAD (Fig. 2A) and then analyzed the correlation between TACR2 expression levels and immune cell content (Fig. 2B). We found that TACR2 expression levels positively correlated with MAST cells (Fig. 2C: COR = 0.22, *P* < 0.001). TACR2 expression levels negatively correlated with M2 macrophages (Fig. 2D: COR = − 0.17, *P* < 0.001). These trends suggest that TACR2 expression is a prognostic protective factor for prostate cancer.

### TACR2 co-expression network

**Fig. 3:**
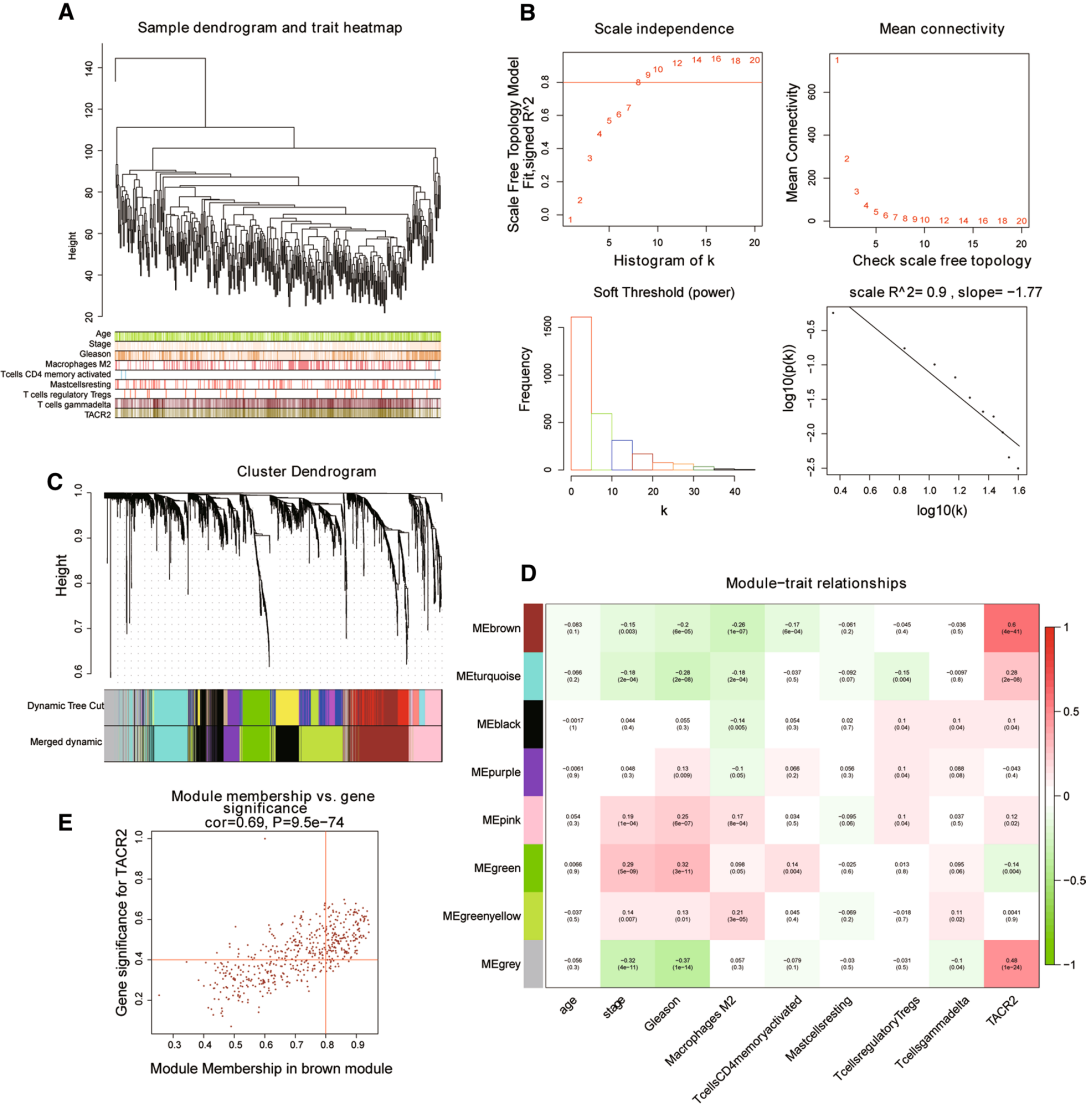
Results of WGCNA analysis in TCGA-PRAD. **A** According to the cutting height = 120,000, 495 samples were included, and the tree diagram of 265 samples was obtained. **B** We built scale-free co-expression networks. The best soft threshold was 5, R-squared was 0.9. **C** A hierarchical cluster tree was constructed, with each leaf representing a gene and each branch representing a co-expression module, and a total of eight co-expression modules were generated. **D** Correlation coefficients between different factors and co-expression modules, where the Brown module (COR = 0.6; *P* = 4E−41) and grey module (COR = 0.48; *P* = 1E−24) had a high correlation with TACR2. The correlation between Brown module membership and gene significance for TACR2 was COR = 0.69; *P* = 9.5e−74

We attempted to construct a co-expression network of TACR2 by analyzing its protein-coding genes and exploring the biological processes involved in TACR2 by enriching the co-expression network’s functions. According to the cutting height = 120,000, 495 samples were included, and a tree diagram of 265 samples was obtained (Fig. 3A). We built scale-free co-expression networks. The best soft threshold was 5, and R-squared was 0.9 (Fig. 3B). A hierarchical cluster tree was constructed, with each leaf representing a gene and each branch representing a co-expression module. A total of eight co-expression modules were generated (Fig. 3C). The Brown module (COR = 0.6; *P* < 0.001) and gray module (COR = 0.48; *P* < 0.001) had high correlations with TACR2 (Fig. 3D). There was a correlation between Brown module membership and the gene significance of TACR2 (Fig. 3E: COR = 0.69; *P* < 0.001).

### TACR2 pathway analysis

**Fig. 4:**
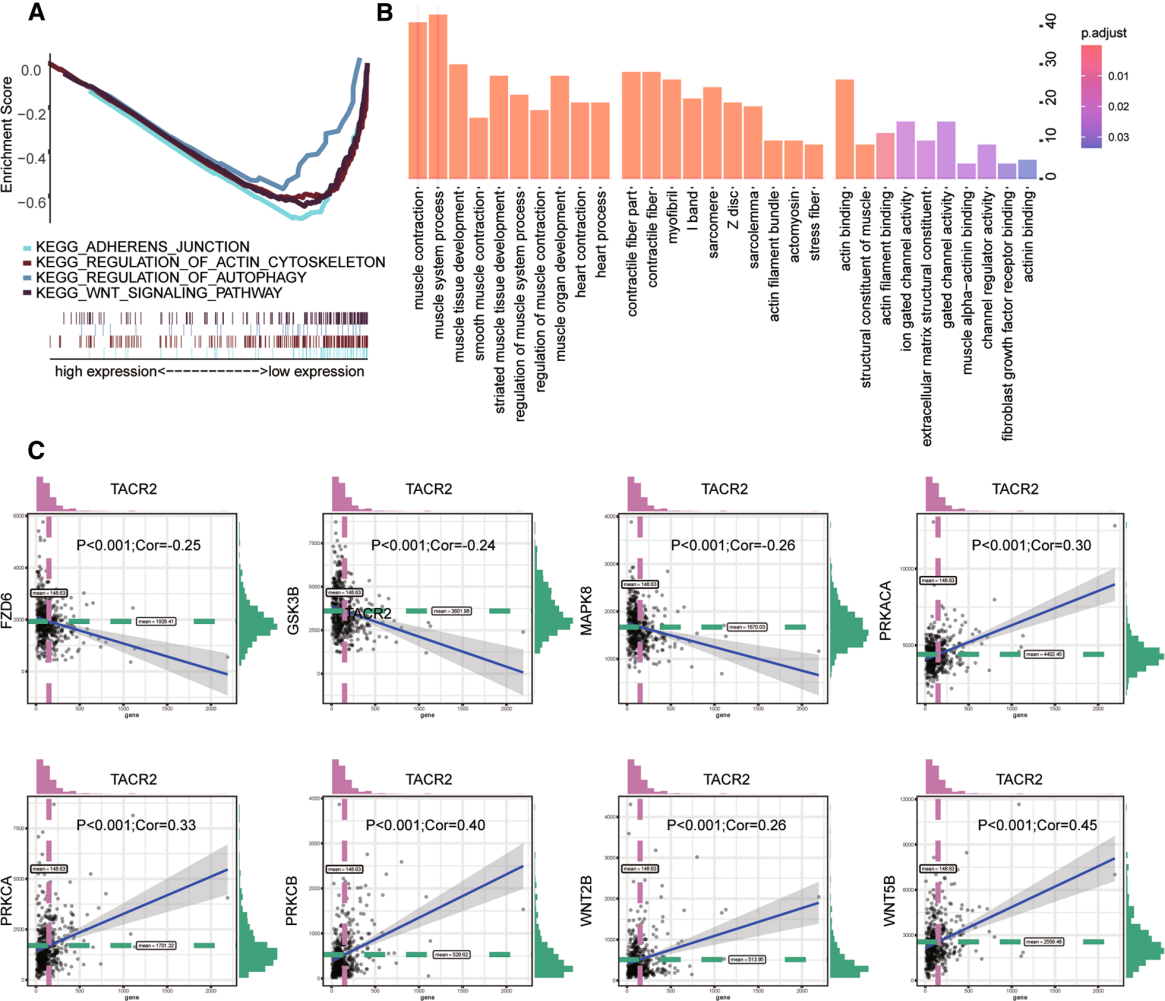
**A** In TCGA-PRAD, TACR2 was enriched in adherent junctions, regulation of actin cytoskeleton, regulation of autophagy, and the Wnt signaling pathway. **B** Biology process analysis of TACR2 in TCGA-PRAD. **C** Correlation between TACR2 and important related genes in the Wnt pathway. TACR2 negatively correlated with FZD6 (*P* < 0.001; COR = − 0.25), GSK3B (*P* < 0.001; COR = − 0.24) and MAPK8 (*P* < 0.001; COR = − 0.26). TACR2 positively correlated with PRKACA (*P* < 0.001; COR = 0.30), PRKCA (*P* < 0.001; COR = 0.33) and PRKCB (*P* < 0.001; COR = 0.40), Wnt2B (*P* < 0.001; COR = 0.26), and Wnt5B (*P* < 0.001; COR = 0.45)

We found that TACR2 is associated with the Wnt pathway in the GSE46602 cohort. We obtained the same result in TCGA-PRAD (Fig. 4A). Then, we enriched the co-expression network of TCAR2. The enrichment results showed that TACR2 is involved in biological processes such as muscle contraction (Fig. 4B). In the pathway analysis, GSE46602 and the TCGA-PRAD cohort confirmed that TACR2 might regulate the Wnt pathway. This pathway affects the progression of prostate cancer. Therefore, we performed a Pearson pathway analysis on the expression of TACR2 and the genes in the Wnt pathway. We found that TACR2 and gene expression levels in the Wnt pathway were also closely related (Fig. 4C).

### Expression of TACR2 and β-catenin in prostate cancer and adjacent normal tissue

**Fig. 5:**
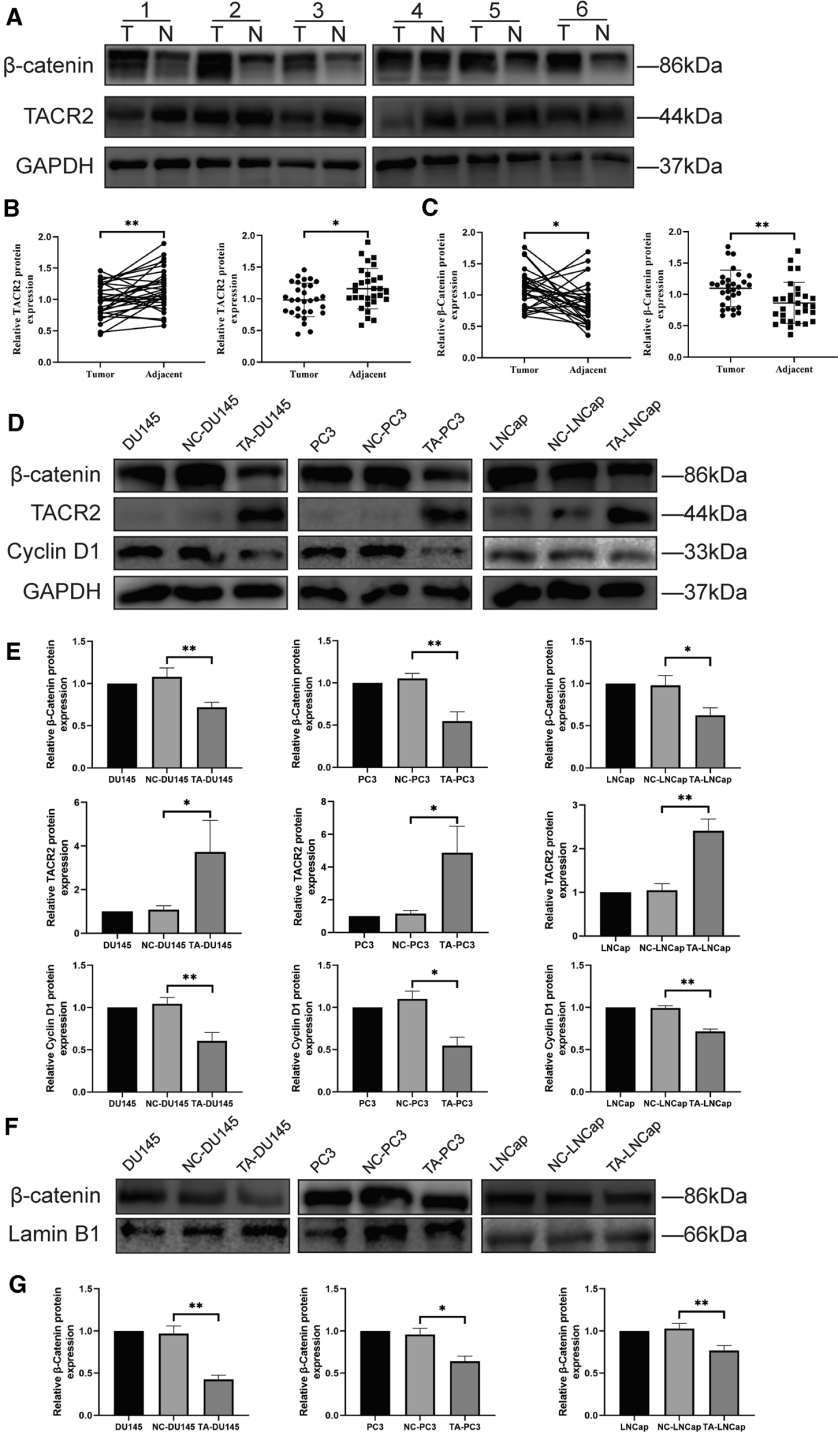
Expression of TACR2 and β-catenin in prostate cancer and prostate cancer cell lines. **A**, **B** The expression of TACR2 protein in prostate cancer tissues (T) and adjacent tissues (N) (*P* < 0.01). **A**, **C** The expression of β-catenin in prostate cancer tissues and adjacent tissues (*P* < 0.01). **D**, **E** After transfection with TACR2 overexpression lentiviral vector and corresponding negative control lentiviral vector in DU145, PC3, and LNCaP, protein levels of TACR2, β-catenin and Cyclin D1 were measured using western blot. **F**, **G** After isolating nuclear proteins, the nuclear protein levels of β-catenin in TA-OE and NC cells were measured using western blot. The samples derive from the same experiment and those blots were processed in parallel

TCGA database analysis showed that transcription levels of TACR2 were significantly different between prostate cancer and adjacent normal tissues. Western blot was used to measure expression levels of TACR2 protein in 30 pairs of prostate cancer and adjacent normal tissues. Consistent with the TCGA database, expression levels of TACR2 protein in prostate cancer tissues were significantly lower than in adjacent tissues (Fig. 5A, B, *P* < 0.01). Next, we found that the expression of β-catenin in prostate cancer tissues was significantly higher than in adjacent tissues (Fig. 5A, C, *P* < 0.01). The clinicopathological data of these 30 patients with prostate cancer was shown in Additional file 1: Table S1.

### Overexpression of TACR2 deactivates the Wnt/β-catenin signaling pathway

To verify the effect of TACR2 on Wnt/β-catenin signaling transduction, we selected hormone-resistant prostate cancer cells (DU145 and PC3) and hormone-sensitive prostate cancer cells (LNCaP) and transfected them with TACR2 overexpression lentiviral vector and corresponding negative control lentiviral vector. Western blot test showed that protein levels of TACR2 in the TA-OE cell lines were significantly higher than in the NC cells (Fig. 5D, E, *P* < 0.01). β-Catenin is the core component of the Wnt/β-catenin signaling pathway; its increased levels suggest pathway activation [21, 22]. Compared with the NC cells, expression levels of β-catenin and Cyclin D1 proteins in the TA-OE cell lines were significantly higher (Fig. 5D, E, *P* < 0.01). After isolating nuclear proteins, we found that the nuclear protein levels of β-catenin in TA-OE cells were significantly lower than that in NC cells (Fig. 5F, G, *P* < 0.01). This evidence suggests that TACR2 overexpression significantly blocks activation of the Wnt/β-catenin signaling pathway.

### Overexpression of TACR2 inhibited cell activity, proliferation, and migration

**Fig. 6:**
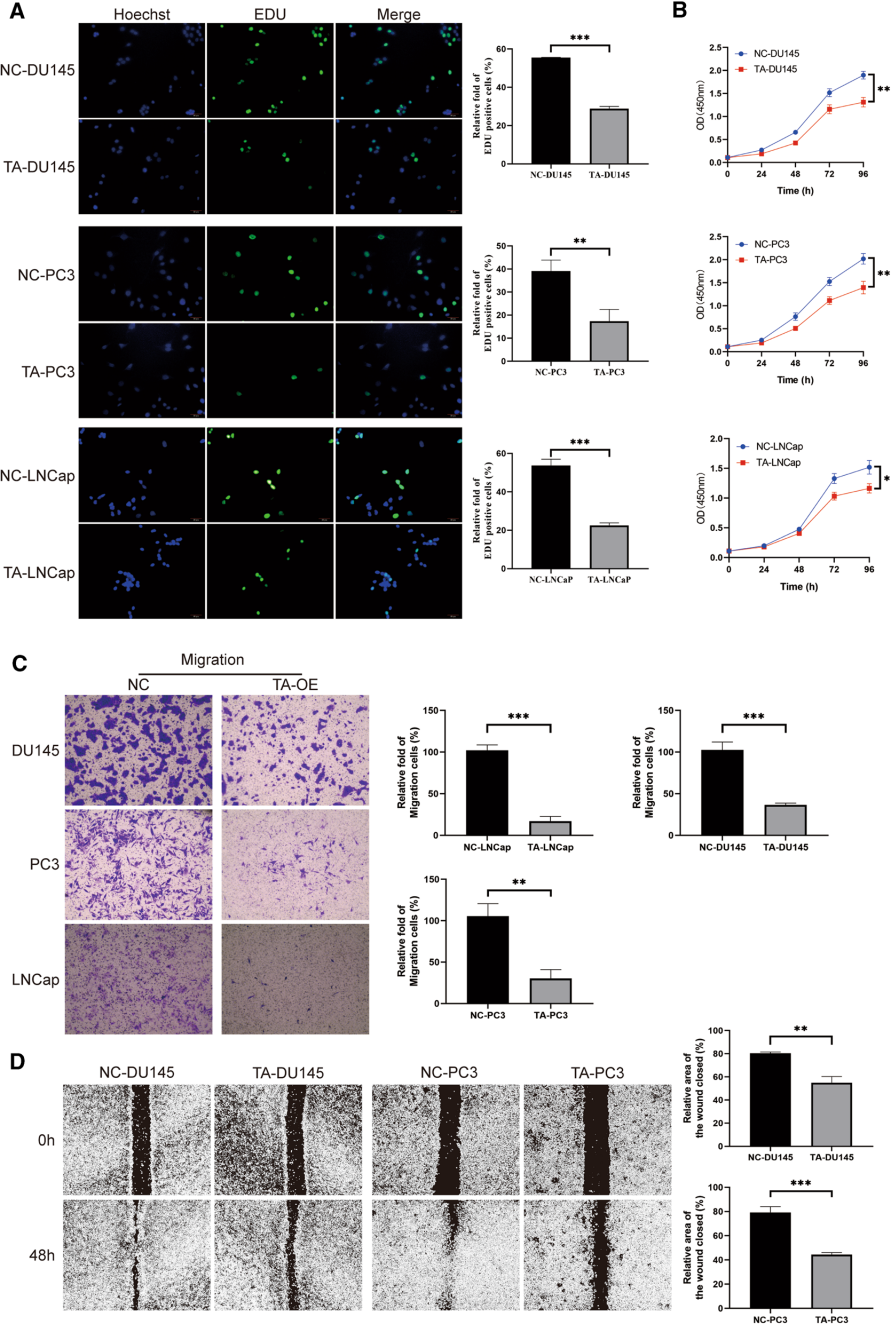
Overexpression of TACR2 inhibited cell activity, proliferation, and migration. **A** The effect of TACR2 overexpression on the proliferation of DU145, PC3, and LNCaP cells were determined using the EdU assay (magnification ×200). **B** Overexpression of TACR2 suppressed cell viability according to the CCK-8 assay. **C** Overexpression of TACR2 inhibited cell migration according to the Transwell assay (magnification ×20). **D** According to the wound-healing assay, overexpression of TACR2 inhibited cell migration in 5637 and T24 cells (magnification ×20)

To further study the biological function of TACR2 in prostate cancer, the EdU test was used to measure the proliferation of TACR2-overexpressing cells. We found that the cells’ proliferation ability was lower than that of NC cells (Fig. 6A, *P* < 0.01). The CCK-8 was used to analyze the activity of TACR2-overexpressing cells. The TA-OE cell lines activity was significantly lower than that of NC cell lines (Fig. 6B, *P* < 0.05). We used a wound-healing migration assay and a Transwell assay to determine the role of TACR2 in prostate cancer cell migration. Transwell assays showed that the cell migration decreased with overexpression of TACR2 (Fig. 6C, *P* < 0.01). Consistently, the wound-healing migration assay showed that the migration ability of PC3 and DU145 decreased significantly after TACR2 overexpression (Fig. 6D, *P* < 0.01).

## Discussion

In recent years, with the popularity of PSA screening and advances in prostate cancer treatment, prostate cancer incidence has been declining or remaining stable; nevertheless, the mortality rate of prostate cancer has not declined [23]. PSA’s limitation as a biomarker for prostate cancer has been widely reported [24, 25]. Therefore, more specific and useful new biomarkers are needed to surpass the limitations of traditional biomarkers. Identifying new biomarkers and clarifying their signal transduction process will reveal the molecular mechanisms of the occurrence and development of prostate cancer. Doing so will help to predict disease outcome and identify better treatment decisions.

TACR2 is involved in a series of biological processes, including controlling intestinal motility, hormone secretion, and visceral sensitivity [9]. The relationship between TACR2 and tumor remains unclear. Using the TIMER database, we found that the expression of TACR2 was significantly downregulated in a variety of cancer tissues, including prostate cancer, clear cell renal cell carcinoma, chromophobe tumor, and papillary renal cell carcinoma. Expression levels of TACR2 significantly correlated with clinical stage, Gleason score, and survival outcomes. Using GSEA, we analyzed the differential pathways between TACR2 high- and low-expression groups of gse46602 and found that the T cell receptor and Wnt pathways were enriched. Therefore, we performed a detailed analysis of these two pathways.

Recent studies showed that tachykinins and their receptors regulate the development, activation, and function of critical immune cells and the immune system. Tachykinins are also involved in immune cell migration, immune cell proliferation, and inflammation regulation with cytokines [26, 27]. We analyzed the correlation between expression levels of TACR2 and the content of immune cells and found that expression levels of TACR2 positively correlated with mast cells and negatively correlated with M2 macrophages. The number of infiltrating mast cells in tumor tissue is closely related to tumor angiogenesis, differentiation, and metastasis. In the prostate cancer microenvironment, mast cells drive adverse events, including tumor invasion, angiogenesis, biochemical recurrence, and metastasis after radical prostatectomy [28–30]. M2 macrophages cannot present antigens. The higher their content, the weaker the patient’s immune response and the worse the outcome [31–33]. These results suggest that TACR2 may regulate the immune cell receptor pathway by changing the immune cell tumor microenvironment and then affecting the occurrence and development of tumor cells. TACR2 is expected to be a target for immunotherapy of prostate cancer.

Next, we analyzed the protein-coding genes of TACR2 to construct its co-expression network and explored the biological process of TACR2 by enriching the function of the co-expression network. The enrichment results showed that TACR2 was involved in muscle contraction and other biological processes. In the pathway analysis, the TCGA-PRAD cohort confirmed that TACR2 might regulate the Wnt signaling pathway. We also found that TACR2 is closely related to the expression levels of critical genes in the Wnt pathway. We measured protein expression in 30 pairs of prostate cancer and adjacent normal tissues. Consistent with the data analysis, the expression of TACR2 protein in prostate cancer tissues was significantly lower, while the expression of β-catenin was significantly higher. Three cell types (hormone-sensitive LNCaP cells, non-hormone-sensitive PC3, and DU145 cells) were selected for TACR2 overexpression. Compared with NC cells, the total protein and nuclear protein levels of β-catenin in TA-OE cells was significantly greater. The protein expression level of Cyclin D1, the downstream target gene of β-catenin, was also consistent with the trend of β-catenin in NC and TA-OE cells. This evidence suggests that TACR2 overexpression significantly blocks the activation of the Wnt/β-catenin signaling pathway. Further cell function experiments showed that overexpression of TACR2 inhibited the activity, proliferation, and migration of prostate cancer cells.

In conclusion, TACR2 is expressed at low levels in prostate cancer tissue and inhibits prostate cancer cells’ migration and proliferation via the Wnt/β-catenin signaling pathway. These findings suggest TACR2 may affect tumor cells’ occurrence and development by changing the content of immune cells in the tumor microenvironment. Nevertheless, the specific mechanisms of TACR2 and the Wnt/β-catenin signaling pathway and the mechanism of regulating the tumor microenvironment require further study. These findings suggest that TACR2 may be a new biomarker and may provide a theoretical basis for prostate cancer treatment.

## Methods

### Statement

The collection of tissue samples was approved by the Ethics Committee of the First Hospital of China Medical University according to the Declaration of Helsinki, and all participants provided written informed consent (approval number, [2018] 2018-190-2). All methods were performed in accordance with the relevant guidelines and regulations.

### Matrix source

The prostate cancer mRNA matrix and clinical information were obtained from TCGA (The Cancer Genome Atlas) database (https://www.cancer.gov/) [34]. We considered 547 prostate samples, including 52 normal prostate samples and 495 tumor samples. We downloaded 36 prostate cancer tissues matrix and the clinical information from GSE46602, the platform for which is the GPL570 Affymetrix Human Genome U133 Plus 2.0 Array [35].

### Pathway analysis

There are few studies on TACR2 at present. To explore the pathways related to TACR2 in prostate cancer, we conducted Gene Set Enrichment Analysis (GSEA) [36]. The GSEA method is used to determine whether a gene set of interest differs significantly in two biological states. We used GSEA to explore functional pathways related to TACR2 expression, grouping according to the median of TACR2 expression. Using a single data set that causes high false-positive results, we performed cross-validation analysis between TCGA-PRAD, and GSE46602.

### TACR2 co-expression mRNA analysis

We found that TACR2 was significantly related to the Wnt pathway. We know that the Wnt pathway is involved in a variety of biological processes in the body. To determine which biological processes TACR2 relates to, we used Gene Ontology (GO) (http://geneontology.org/) analysis to illustrate the biological function of TACR2 co-expressed protein-coding genes [37]. We used the weighted gene co-expression network (WGCNA) method to explore the co-expression factors of TACR2 by constructing a scale-free network [38]. The number of genes in the minimum module was defined as 30. By drawing a correlation heat map between the module and TACR2, we determined the module with the strongest correlation with TACR2. We extracted protein-coding genes with Pearson correlation coefficients > 0.4. GO analysis of these genes identified the most relevant biological functions and molecular results of TACR2. We did not directly screen based on the Pearson correlation coefficient of TACR2 and other protein-coding genes; instead, we first determined its co-expression module before the screening. Such a screening strategy assumes that co-expression modules usually have similar biological behaviors. The most relevant co-expression module of TACR2 can be obtained using this method, determining the most relevant biological function.

### Immune cell proportion correlation

There is tachykinin-mediated modulation of the immune response [39]. Therefore, we attempted to determine associated immune processes by measuring prostate cancer tissues’ immune cell content. We ran the CIBERSORT algorithm and determined the immune cell proportion based on the bulk tissue gene expression matrices. LM22 is a gene signature matrix that defines 22 immune cell subtypes obtained from a website (https://cibersort.stanford.edu/).

### Clinical specimen collection

From January 2020 to December 2020, we performed radical prostatectomy on prostate cancer patients according to EAU GUIDELINES ON PROSTATE CANCER, and 30 pairs of prostate cancer tissue samples and adjacent normal prostate tissue samples (> 1 cm from the tumor) were collected at the First Hospital of China Medical University (Shenyang, China). No patients received endocrine therapy, chemotherapy, or radiotherapy before radical prostatectomy. All tissues were examined histologically. Prostate cancer tissue samples had high-density cancer foci, and no cancer foci were found in the adjacent normal prostate tissue samples. Tissue samples were stored at − 80 °C before use.

### Cell lines and reagents

The human prostate cancer cell lines, PC3, DU145, and LNCaP Clone FGC, were obtained from the National Collection of Authenticated Cell Cultures (Shanghai, China) and were STR certified. PCR was used to detect Mycoplasma in the culture medium, and the passage time of the cells was not more than 6 months. The cell lines were cultured in RPMI 1640 (HyClone, USA) supplemented with 10% fetal bovine serum (FBS, Gibco) and grown at 37 °C, 5% CO_2_. The antibodies included TACR2 (Proteintech, 25270-1-AP), beta-Catenin (Proteintech, 51067-2-AP), GAPDH (Cell Signaling Technology, 5174S), Cyclin D1 (Cell Signaling Technology, 55506S), Lamin B1 (Cell Signaling Technology, 13435S).

### Construction of cell lines with TACR2 overexpression

PC3, DU145, and LNCaP cells were seeded in 6-well or other plates and were transfected 24 h later. Genechem (Shanghai, Genechem) was commissioned to design and synthesize the TACR2 overexpression lentiviral vector and the corresponding negative control lentiviral vector. Three cell lines were transfected according to the manufacturer’s protocol. The stable negative control (NC) and TACR2 overexpression (TA-OE) cell lines were established in a puromycin-containing medium.

### Western blot analysis

The total proteins were extracted from tissues and cell lines using RIPA lysis buffer (containing protease and phosphatase inhibitors). Nuclear protein was extracted using the Nuclear and Cytoplasmic Protein Extraction kit (Beyotime). Protein concentration was determined with bicinchoninic acid assay (Beyotime Institute of Biotechnology). The denatured protein sample (40 µg/lane) was added to the 10% sodium dodecyl sulfate–polyacrylamide gel for electrophoresis (140 V, 60 min), then separated and transferred to the polyvinylidene fluoride membrane (340 mA, 90 min). Subsequently, membranes were blocked in a sealed container with 5% non-fat milk (37 °C, 1 h) and incubated with anti-TACR2 (1:500), anti-β-catenin (1:5000) or anti-GAPDH (1:1000) primary antibodies overnight in 5% fat-free milk at 4 °C.

### EdU assay

The stable cells were seeded on 24-well plates. According to the manufacturer’s instructions, EdU (BeyoClick™, EDU-488, China) reagent was added to the medium at the ratio of 1:1000. According to the manufacturer’s protocol, the culture was continued at 37 °C for 2 h; after labeling, complete the following experimental steps. Finally, the number of proliferating cells was counted under the fluorescence microscope (Olympus Corporation, Japan).

### Cell viability assay

The stable PC3, DU145, and LNCaP cells were seeded into 96-well plates at the density of 5 × 10^3^ cells/well. Cell Counting Kit-8 (CCK-8) (Bimake, USA) solution was added to each well to a final concentration of 0.5 mg/ml and incubated at 37 °C for 1 h. The absorbance was measured at 450 nm using a plate reader (Model 680; Bio-Rad Laboratories).

### Transwell assay

Transwell chambers without matrix were used for cell migration tests. We plated 200 µl of serum-free medium containing stable cells (1 × 10^5^) into the upper chambers (Corning, NY, USA) of 24-well plates, and 600 µl medium (10% FBS) was plated into the lower chamber. After 48 h of incubation, the cells under the membrane were fixed with 4% paraformaldehyde for 10 min and stained with crystal violet stain for 10 min. The remaining cells in the upper chamber were removed with cotton swabs. The migration of cells on the surface under the membrane was measured using an inverted light microscope, and the migration efficiency was calculated using ImageJ.

### Wound-healing migration assay

Wound-healing tests were used to measure cell migration. Stable PC3 and DU145 cells were seeded on 24-well plates. When 90–100% confluence was achieved, a 200-µl sterile pipette was used to create a linear scratch. After washing with a serum-free medium, the cells were cultured for 48 h. Cell images at 0 and 48 h after injury were recorded under a microscope.

### Statistical analysis

GraphPad Prism version 8.0 (GraphPad Software Inc., USA) was used for statistical analyses. The data of at least three independent experiments were expressed as mean ± standard deviation. The differences between groups were analyzed using Student’s *t*-test. Statistically, significance was set at *P* < 0.05. Pearson coefficients > 0.4 were considered significant. We used R-version 3.6.3 for all the R package statistical analyses.

## Supplementary Information

**Additional file 1: Table S1.** The clinicopathological data of 30 patients with prostate cancer.

## Data Availability

Publicly available datasets were analyzed. These data can be found in the following locations: the datasets TCGA-PRAD for this study can be found at http://cancergenome.nih.gov/. The datasets GSE46602 and for this study can be found at http://www.ncbi.nlm.nih.gov/geo/.

## Abbreviations

TACR2: Tachykinin receptor 2
PSA: Prostate-specific antigen
TCGA: The Cancer Genome Atlas
WGCNA: Weighted gene co-expression network
GO: Gene Ontology
NC: Negative control cell lines
TA-OE: TACR2 overexpression cell lines
CCK-8: Cell Counting Kit-8
