# Supplementary material for: TACR2 is associated with the immune microenvironment and inhibits migration and proliferation via the Wnt/β-catenin signaling pathway in prostate cancer
Source: Cancer Cell Int. 2021 Aug 7;21:415. doi: 10.1186/s12935-021-02126-0 (PMC8349497; doi:10.1186/s12935-021-02126-0)
Supplement: Supplementary file 1 — Additional file 1: Table S1. The clinicopathological data of 30 patients with prostate cancer. [file 12935_2021_2126_MOESM1_ESM.docx]

**Supplementary Table 1 Clinicopathological data of 30 patients with prostate cancer**

| case | age (years) | preoperative PSA (ng/ml) | Gleason score | TNM stage (AJCC, 2017) | number of positive lymph nodes / lymph nodes | positive surgical margin | prostate volume (ml) | preoperative endocrine therapy |
| --- | --- | --- | --- | --- | --- | --- | --- | --- |
| 1 | 62 | 19.5 | 3+4=7 | T2cN0M0 | L: 0 / 3; R: 0 / 2 | No | 38.6 | No |
| 2 | 65 | 5.8 | 3+3=6 | T2cN0M0 | L: 0 / 3; R: 0 / 8 | No | 16.3 | No |
| 3 | 61 | 15.9 | 4+3=7 | T3aN0M0 | L: 0 / 4; R: 0 / 6 | Yes | 16.2 | No |
| 4 | 69 | 10.0 | 4+3=7 | T2aN0M0 | L: 0 / 6; R: 0 / 1 | No | 54.8 | No |
| 5 | 69 | 12.1 | 4+3=7 | T2cN0M0 | L: 0 / 2; R: 0 / 5 | No | 29.6 | No |
| 6 | 48 | 27.9 | 3+4=7 | T2cN0M0 | L: 0 / 8; R: 0 / 5 | No | 39.0 | No |
| 7 | 71 | 39.0 | 3+4=7 | T2aN0M0 | L: 0 / 7; R: 0 / 8 | No | 150.9 | No |
| 8 | 66 | 20.9 | 4+3=7 | T2cN0M0 | L: 0 / 11; R: 0 / 6 | No | 34.7 | No |
| 9 | 75 | 16.1 | 3+4=7 | T2cN0M0 | L: 0 / 6; R: 0 / 4 | No | 28.6 | No |
| 10 | 65 | 17.0 | 4+3=7 | T1cN0M0 | L: 0 / 3; R: 0 / 3 | No | 64.2 | No |
| 11 | 74 | 25.0 | 5+4=9 | T3bN0M0 | L: 0 / 2; R: 0 / 5 | Yes | 35.9 | No |
| 12 | 74 | 37.7 | 4+3=7 | T3bN0M0 | L: 0 / 6; R: 0 / 5 | Yes | 26.2 | No |
| 13 | 71 | 12.7 | 3+4=7 | T2bN0M0 | L: 0 / 10; R: 0 / 4 | No | 41.9 | No |
| 14 | 66 | 20.2 | 4+3=7 | T2cN0M0 | L: 0 / 6; R: 0 / 5 | No | 15.1 | No |
| 15 | 63 | 19.8 | 3+4=7 | T2bN0M0 | L: 0 / 4; R: 0 / 4 | No | 28.4 | No |
| 16 | 67 | 21.4 | 3+3=6 | T2cN0M0 | L: 0 / 11; R: 0 / 12 | No | 45.9 | No |
| 17 | 62 | 37.8 | 4+3=7 | T3aN0M0 | L: 0 / 6; R: 0 / 7 | Yes | 36.2 | No |
| 18 | 68 | 32.4 | 4+5=9 | T2cN0M0 | L: 0 / 10; R: 0 / 9 | No | 21.6 | No |
| 19 | 64 | 21.1 | 4+3=7 | T2bN0M0 | L: 0 / 5; R: 0 / 5 | No | 20.1 | No |
| 20 | 62 | 39.6 | 5+4=9 | T2cN1M0 | L: 1 / 7; R: 1 / 6 | No | 57.4 | No |
| 21 | 55 | 11.3 | 5+4=9 | T3bN1M0 | L: 0 / 4; R: 1 / 4 | Yes | 59.0 | No |
| 22 | 69 | 44.9 | 3+4=7 | T2cN0M0 | L: 0 / 5; R: 0 / 4 | No | 32.3 | No |
| 23 | 71 | 11.3 | 5+4=9 | T4N1M0 | L: 0 / 8; R: 1 / 9 | Yes | 38.5 | No |
| 24 | 67 | 9.2 | 4+3=7 | T2cN0M0 | L: 0 / 14; R: 0 / 16 | No | 33.1 | No |
| 25 | 63 | 24.6 | 4+5=9 | T3aN0M0 | L: 0 / 1; R: 0 / 1 | Yes | 27.2 | No |
| 26 | 63 | 9.1 | 3+4=7 | T2cN0M0 | L: 0 / 3; R: 0 / 3 | No | 14.5 | No |
| 27 | 50 | 37.2 | 4+3=7 | T2bN0M0 | L: 0 / 5; R: 0 / 4 | No | 43.4 | No |
| 28 | 70 | 3.0 | 3+5=8 | T2aN0M0 | L: 0 / 9; R: 0 / 9 | No | 35.0 | No |
| 29 | 69 | 20.1 | 3+3=6 | T2cN0M0 | L: 0 / 4; R: 0 / 4 | No | 21.9 | No |
| 30 | 65 | 13.5 | 3+5=8 | T2cN0M0 | L: 0 / 7; R: 0 / 6 | No | 46.0 | No |
